# Two “Popes” to Speak for the World: The Pope and the United Nations Secretary General in World Politics

**DOI:** 10.1080/15570274.2017.1392712

**Published:** 2017-11-13

**Authors:** Jodok Troy

Despite recent studies on leadership, the discipline of International Relations is still reluctant to engage in studies of individual agency in the international structure.^1^ Two prominent examples are the leader of the Catholic Church, the pope, and the United Nations (UN) Secretary-General (UNSG). Neither of them is a leader in control of considerable hard power, yet both exemplify the puzzle of how institutions, individuals, and moral authority relate in leadership. I argue that it is a combination of individuals in institutions that leads to unexpected and unintended effects such as the evolution of the papacy and the UNSG as instances of moral authorities. While pointing out their potential for moral leadership, this article presents a conceptual framework of how to perceive the Pope and the UNSG in world politics. The article unfolds in three sections: in the first, I look at the potential of comparing the two positions in terms of moral leadership and their emphasis of the common good. In a literature review, I then outline the current state of the literature on the two positions and what it misses. The remainder of the paper proposes a conceptual framework on how the two positions fit into the current literature and what promising future research for International Relations it conceals.

## Servant of God, Servant of Governments

In many regards, both positions, the papacy and the UNSG, represent an anachronism. The individuals that represent them are elected in dubious ways, influenced by variables hard to grasp, and both are heirs of political developments that shaped the establishment of positions and institutions that have long since changed. Nevertheless, the pope and the “secular Pope,” the UNSG, are two individuals whose voices are given particular attention in world politics. They are quite similar as both depend on the character of the office holder and the institution. It is thus not only about authority and autonomy of the organization arising out of delegated, moral, and expert authority (Barnett and Finnemore 2004, 20–29; Hall 1997). Rather, like executive heads,^2^ the Pope and the UNSG are in authority and an authority themselves (see also Barnett and Finnemore 2004, 25). Eventually, such positions lead to good leadership which is “valued as a moral necessity” (Burns 2003, 2).^3^

‘Vous êtes mon homologue laïque,” Pope Pius XXII told UNSG Dag Hammarskjöld (Lipsey 2013, 153), who himself considered the UN a “secular church” (Foote 1962, 47).^4^ Pope John XXIII (1963) addressed in his encyclical *Pacem in Terris* “all men and women of goodwill” and Hammarskjöld argued that “all men and women of good will can influence the course of history in the direction of the ideals expressed in the Charter” (Foote 1962, 45). In his most recent encyclical, Pope Francis (2015) even speaks to “every person living on this planet.” Putting forward such broad claims suggests that both individuals perceive themselves as moral authorities, working for the common good.

Moral authority invoking the common good is not only a publicly called for demand, but a necessity of human conduct in a globalized world. The world is facing a growing “enlargement of human expectations” as UNSG Cuéllar (1995, 168) called it. This makes the pope and the UNSG disputed but appealing figures of moral authority. While there is no way to foresee the end of the current papacy, Francis seems to be an indicator that supports the main thesis of this article: it is the combination of institutions and individuals that shapes their positions and establishes the power of moral leadership that eventually transforms institutions (Franco 2013a, 2013b; Ivereigh 2015).

**Table:** 

Popes since 1945	UNSGs since 1945
Pius XII (1939–1958)	Trygve Lie (1946–1952)
St. John XXIII (1958–1963)	Dag Hammarskjöld (1953–1961)
Paul VI (1963–1978)	U Thant (1961–1971)
John Paul I (1978)	Kurt Waldheim (1972–1981)
St. John Paul II (1978–2005)	Javier Perez de Cuellar (1982–1991)
Benedict XVI (2005–2013)	Boutros Boutros-Ghali (1993–1996)
Francis (2013–)	Kofi Annan (1997–2006)
	Ban Ki-Moon (2007–2016)
	António Gutierrez (2017–)

There are extensive studies on the power and the moral authority of the UNSG (Chesterman 2007; Kille 2007, 2006), less so and mainly historical ones on the pope (Coppa 1998, 2008, 2014; Hall 1997; Riccards 1998). This is surprising, as research points out that via their leaders, institutions can have unexpected and unintended effects beyond their formal codified mission statement (Barnett and Finnemore 1999). This, however, is only possible if institutions are, first, assumed to be moral agents within a social practice and, second, if the leaders’ followers are taken into account of the potential and extent of moral authority (Kellerman 2008): “The very notion of an individual moral agent presupposes the existence of a collective practice with its associated ethic embedded in it” (Frost 2003, 98 original in italics; see also Frost 1996). Agency, then, takes shape in and due to sociality. Nonetheless, modest emphasis has been placed on leadership and transformative action. Leaders engage in transformative action if they override existing structures and create something unusual based on their agency (Menaldo 2013; Saunders 2009).^5^

Many definitions of leadership focus on traits-based leadership. They define a leader as “someone who helps a group create and achieve shared goals” (Nye 2008, 18). Often it is charisma that drives moral authority as charismatic authority is “a rule over men, whether predominantly external or predominantly internal, to which the governed submit because of their belief in the extraordinary quality of the specific *person*” (Weber, Gerth, and Mills 1946, 295). Likewise, role theory and micro foundations of behavioural International Relations hold that the positions have changed because key individuals have changed the role of that position in the institution (Harnisch, Frank, and Maull 2011; Walker, Malici, and Schafer 2011; see also Hudson 2005).

Moreover, definitions of leadership distinguish between leadership and executive tasks to “differentiate the responsibilities and authority.” However, executive leadership is about “determining an organization’s goals and mobilizing constituents to achieve those goals” (Schroeder 2014, 3). Most broadly, the literature on leaders (e.g. Byman and Pollack 2001; Chiozza and Goemans 2011; Isaak 1975) and leadership (e.g. George 1969; Hermann 1980) point out particular moments when the impact of individual leaders on the course of events is most likely. This is when political institutions are unstable, young, in crisis, collapse, institutional constraints are limited and the issue or situation is peripheral, unusual, or ambiguous (Mingst and Arreguín-Toft 2013, 180–181).

## Institutions, Agency, and the Pope’s and UNSG’s Moral Leadership

I argue that a combination of individuals in institutions leads to the influence and possession of moral authority beyond constitutional arrangements and external structural constraints and opportunities. A parallel look at the pope and the UNSG facilitates such an understanding of agency and leadership. While stressing a comparative perspective (Helms 2012), this provides additional insights into ontological and epistemological developments and produces more general results on moral leadership.^6^

There are two basic caveats to this argument. First, comparing the pope and the UNSG brings up shortcomings in terms of structural comparability and technicalities. However, apart from choosing them because of their normative attributes, both can be compared because of the nature of the organizations in which they are embedded.^7^ The character of agents, institutional constraints, and external effects influence the two institutions and their individual agency. Whereas in-house constraints (e.g. the Curia, the Security Council) can candidly be located, the impact of external influences is hard to evaluate. Second, despite the similarities between the pope and the UNSG, there are limiting parameters of a comparison in the formulation of their power (i.e. political and religious leader vs. administrative official).^8^ The most important one is that the pope, the “Vicar of Christ” and absolute monarch is, ipso facto, leader of the Catholic Church. The UNSG is chief administrative officer of the UN. Even more so, his potential for moral leadership is constrained by structural forces rooted in a world of states (Thakur 2017, 11).

One might argue that the UNSG is an administrative officer and mediator, whereas the pope is a religious and political leader. However, popes and UNSGs have been engaged in in-house (i.e. “catholic”) and external (i.e. “political”) mediation. The political engagement of John Paul II during the Cold War comes to mind. But also his successors, Benedict XVI (e.g. on the cultural foundation of Europe) and Francis (e.g. on improving Cuban–US relations) (Álvarez 2014) intermingled in international politics beyond rhetorical appeals. Another example is Pope Francis’ attempt to escape existing political rivalries, avoiding mirroring the formerly East–West tensions. Instead, he is shifting the focus on geopolitics to Latin America like Pope John Paul II did for Eastern Europe (Flamini 2014; Levine 2016). A particular case in point is Francis’ allegedly “giving up” on human rights and referring instead more to social justice, structural problems, and collective solutions that individual rights probably are not able to solve (Moyn 2015). Another example is the prevailing international trend of enforcing responsibility and accountability (Ainley 2008; Sikkink 2011). This trend has been pushed (e.g. by Ghali and Pius XII) and restrained (e.g. by Hammarskjöld and Francis) by popes and UNSGs alike.

### Individuals in Institutions

The literature on leadership does not address that what and how leaders do something is as important as what they are and what resources they command. Whereas “[p]ower does not equal leadership” (Nabers 2011, 88), the opposite is also true: leadership does not equal “hard” power. Examples are John Paul II and Dag Hammarskjöld. In both cases, it was also their charisma which led to transformative leadership. Their performance did not only change the institution due to their delegated, expert, and moral authority. Their charisma became the impetus for institutional change and policy outreach and, ultimately, account for transformative leadership. This is the case for Hammarskjöld’s efforts (e.g. the independent international civil service), John XXIII’s initiatives (e.g. the initiation of the second Vatican Council), or John Paul II’s public stance on matters of the Cold War (e.g. the help to initiate protests against communist regimes) (Weigel 1992).

Popes and UNSGs laid out strategic plans and visions, yet their implementing strategies differed widely. Hammarskjöld had a vision of an independent Secretariat and civil service which he implemented (Childers and Urquhart 1994; Hammarskjöld 1960; Orford 2011, chapter 2; Sending 2015, 47–53; Sinclair 2015). John XXIII’s vision of peace and engaging in the crises in the nuclear age is another example (Flamini 1980; Zizola 1978). Some chose follower-oriented strategies, while others chose opposition-oriented strategies (i.e. mobilizing those opposed to the vision). Some of them exercised self-restraint while working on their vision (e.g. Hammarskjöld changed the organization of the Secretariat, John XXIII initiated the Second Vatican Council), some did not (e.g. Boutros-Ghali, John Paul II). Some accompanied their implementation strategies with institutional and administrative changes (e.g. Hammarskjöld, John Paul II), some not (e.g. Boutros-Ghali, John XXIII).^9^

Studies on the impact of individuals reveal limits when it comes to the pope’s and the UNSG’s host institutions. Both institutions, the Holy See and the UN, are not “new.” Consider the cases of Hammarskjöld and John XXIII: the UN Secretariat at that time was young and institutional constraints were limited (e.g. via the Security Council at the beginning of the term). The conditions Hammarskjöld encountered where unusual and ambiguous (e.g. the joint intervention of France and Great Britain over the Suez Canal crisis and the subsequent installation of the first UN peacekeeping mission). The same is true for the tenure of John XXIII. Although the papacy was not young, unstable, or in a state of crisis, the institutional constraints (e.g. via the Curia) were rather limited. What is more, in the Cuban Missile Crisis John XXIII encountered an unusual situation that enabled him to mediate between Khrushchev and Kennedy (Flamini 1980).^10^ The same circumstances enabled U Thant to assist in the Cuban Missile crisis by establishing a communication line between the great powers (Dorn and Pauk 2009).

Looking at the pope and the UNSG demonstrates the lacuna in the literature on leadership of individuals in institutional contexts and their social setting (Battilana 2006; Helms 2014). Although few studies pay attention to the two positions, “[d]ecision making does matter” (Hagan 2001) and “who leads matters” (Hermann et al. 2001). This is even more the case regarding the pope and the UNSG (Cox 1969; Kille 2006; Kille and Scully 2003). Popes and UNSGs, however, might be good “statesmen” and leaders only in the capacity of their offices in the Holy See and in the UNSG Secretariat (Claude 1971, 210). Studies on the UNSGs point out that personal leadership style is paramount (Kille 2006) but principal-agent theories are offering too less of an explanation of the autonomy of UNSGs (Karns 2012).

The papacy and the UNSG Secretariat are potentially shaped by their office holders, internal factors of the institutions, and by variables outside the immediate scope and influence of the institutions and their representatives. Popes often have little international experience and tend to depend on their secretaries of state. The UNSG, on the other hand, is a professional diplomat and servant of many masters. In both cases it is also the “rational-legal authority that [International Organizations] embody also gives them power independent of the states that created them and channels that power in particular directions” (Barnett and Finnemore 1999, 699).

Possessing agency, that is possessing internal powers and capacities (Barnes 2000, 5), is not only based on the pope’s and the UNSG’s weak material and institutional power (i.e. head of state, administrational officer, agenda setter, etc.). It is also based on their normative power provided by their institutions. Claude (1971, 191) observed that while there may be powerful members (such as in the Curia or the Security Council) of international organizations, the staff headed by a secretary or secretary general “*is* the organisation” (see also Mathiason 2007). As such, both positions and the “normative power” they entail matter in world politics as they are prone for moral leadership.

### Moral Leadership

The similarity between the pope and the Secretary-General illustrates, first, how both exercise their posts out of a combination of weak material capability and normative power. Focusing on the normative role of power adds another facet to the discussion on power beyond military capabilities. Evaluating the moral power of the pope and the UNSG takes into account “the socially diffuse production of subjectivity in systems of meaning and signification” (Barnett and Duvall 2005, 43). Second, in recognizing the similarity between the two, the nature of institutions and their office holders are evidence of a mutual relationship of secular and religious world political representation and normative claims therein (Agensky 2017; May et al. 2014; Wilson 2010). Third, looking at them illustrates that both struggle between policy positions of interventionism and restraint.

In representing transnational institutions,^11^ the pope and the UNSG claim to speak for the world’s population. The UNSG speaks in his legal right as chief administrative officer as set up in the Charter (United Nations, art. 1,4), to “be a centre for harmonizing the actions of nations”. In the words of the first UNSG Lie (1954, 88), he is a “spokesman,” for the world interest (see also Chesterman 2015, 506). As such, he has the responsibility to heighten international awareness as UNSG Boutros-Ghali (1996, 88) termed it. The pope, on the other hand, is “the Vicar of Christ, and the pastor of the universal Church on earth” (Code of Canon Law, Can. 331) and “obtains full and supreme power in the Church” (Code of Canon Law, Can. 332 §1). He acts on the grounds that “the Church believes she can contribute greatly toward making the family of man and its history more human” (Second Vatican Council 1965, Art. 40)—a statement similar to the spirit of the Charter of the UN. In any case, each of them has the “constitutional licence to be as big a man as he can” (Claude 1971, 211)—they are potentially moral leaders.

In a globalized world political leadership requires a “bold and noble vision for the community” where the UNSG must have the “elusive ability to make others connect emotionally and intellectually to a larger cause that transcends their immediate self-interest” (Thakur 2006, 333). This is not least since the UN is framed as a “focal point of an emerging ‘world-oneness’” (Pedersen 2010, 339). This is equally true for the Catholic Church, which already in its name proposes to be a unifying authority. Nevertheless, both institutions are representative of the growing pluralization of their constituency on the global scale—religious and secular, modern and anti-modern (e.g. Berger 2014). Moreover, neither of the two commands any traditional hard material power. However, it is not the “lack of an army to command, but [the] lack of a party to lead and a body politic to rally behind” (Claude 1971, 207–08). Looking at single individuals illustrates that they build their office on a vision of the common good and a “body politic to rally behind.”

What is presented as crucial in world politics by the pope and the UNSG as moral leaders is the paramount value both attribute to peace and human rights (e.g. Claude 1996; Matlary 2001). This can be seen by the inter-Christian formulation of the 1948 universal *Human Rights Declarations* and the declaration of the later Popes John XXIII and Paul VI (Nurser 2005, 172, FN 45; Glendon 2001, 132, 217). Eventually, their rhetorical engagement to foster peace and human rights propelled their practical engagement in world politics more broadly.

## The Pope and the UNSG in World Politics

The pope and the UNSG demonstrate executive leadership and entrepreneurial power (Haack and Kille 2012; Kille 2006) by deploying “ideas and information to produce significant structural change” (Goddard 2009, 251). The two institutions and their leaders are “self-directed actors” (Oestreich 2012) in possession of autonomous agency. This aspect also offers insights to international political conduct in focusing on moral stewardship under structural constraints. To give one example: Why is it that the “silent diplomacy” of Hammarskjöld was not carried on to this extent by his successors (Ask and Mark-Jungkoist 2005; Fröhlich 2008; Jones 2004; Jordan 1983; Stahn and Melber 2014), let alone that it became a part of the institutional structure? In a similar way, the shuttle diplomacy of Pope John Paul II, lobbying for political transformation via a conception of anti-politics,^12^ was not carried on by his successor (see also Christiansen 2006).

International leadership, as pointed out above, is about particular individuals in particular institutions (Frost 2003, 84), the ideas they promote, and how they attribute and give the office meaning by those ideas. The rhetorical emphasis of international law as a guarantor for international order is something both leaders emphasize (Johnston 2003; Pontifical Council for Justice and Peace 2004, para. 434). The two individuals act in world politics not on behalf of a particular nation but for collectives and thus transcend inter-national and intra-national boundaries.^13^

Collective practices are an overlooked element that ground themselves not only on the formal, constitutional practice, but on elements that go beyond such a practice.^14^ The question, for example, is not only why John Paul II contributed to the downfall of Communism, but rather how he managed to do so at all and how his actions influenced the conceptualization of the papacy. Inquiring such questions not only provides insights to the papacy and the UNSG but likely also produce general outputs as they are entrepreneurs and followers of normative trends. The pope and the UNSG are nuances in the split between conservative and liberal ideals in world politics. The persistence of nationalism, the “rise of the rest,” and great power aspirations are evidence for prevailing conservative paradigms. Aspirations for global governance, development of principles in favour of human rights such as the *Responsibility to Protect*, cosmopolitanism and the focus on world society are evidence for the latter (Bernstein and Pauly 2007; Meyer et al. 1997; Phillips 2013). The UN Charter always struggled between conservative and liberal poles and so does the papacy, particularly since the end of the Second World War.

“Papal Interventionism” (Walsh 2000) is nothing new. Protecting “fundamental rights of individuals and the rights of people in their quest for authentic self-determination” (Araujo 2007, 368) led the popes to harsh criticism of military interventions (e.g. John Paul II on Operation Desert Storm and Iraqi Freedom). At the same time, this rhetorical emphasis of the popes led the Church to rethink just war theory (Heft 2011) and increased the approval rate of humanitarian interventions. This is evident in the speeches of the UNSGs and popes since the end of the Second World War. Pope Pius XII, for example, could not remain neutral any longer during the Cold War (Kent 2002) and neither could the UNSGs. Eventually, both individuals have been entrepreneurs and followers of international norms, engaging in their life cycles (Finnemore and Sikkink 1998), either pushing or hindering them.

This conceptual framework illustrates that the nature of institutions and their office holders are evidence for a mutual relationship between secular and religious world political representation and normative claims therein. The values and ideas that secularism wanted to ban influence how we live in this global age (Thomas 2010). Religion and politics have always been intermingled and mutually constitutive (Agensky 2017; Hurd 2008; Thomas 2014; Walzer 2007, 147–67). The UN is a case in point where religious norms diffuse into the secular sphere via institutional and individual translation (Bettiza and Dionigi 2014; Dionigi 2016). The “secular church” UN (Lash 1962, 543) can act as an institutional translator, rephrasing religious norms and ideas into secular language. All the same, the UN reaches with its own agenda based on the Charter and subsequent texts into the sacred realm (Barnett and Stein 2012; Luoma-aho 2011).^15^ The UN stands, as Hammarskjöld once remarked, “outside—necessary outside—all confessions but it is, nevertheless, an instrument of faith. As such, it is inspired by what unites and not by what divides the great religions of the world” (Foote 1962, 56–57).^16^

## Conclusion

This article argued, using the example of the pope and the UNSG, that institutions can have effects via their leaders beyond their formal mission statement, often translated as moral leadership. If those individuals link agency and responsibility, they can outgrow constitutional arrangements and external constraints. It is a combination of individuals in institutions that leads to unexpected and unintended effects such as the evolution of the papacy and the UNSG as instances of moral authorities and sometimes even transformative leadership. This requires future empirical research to bolster those preliminary and explanatory propositions presented in this conceptual framework.

## Notes

1. E.g. Horowitz, Stam, and Ellis (2015), Saunders (2014), Sandal (2017), Byman and Pollack (2001).

2. I am aware that, in a formal sense, neither the UNSG nor the pope is an “executive officer.”

3. Regardless the demand for a global leader, in case of the UNSG, this demand “has not generally been matched by a supply of high-quality candidates” (Chesterman 2015, 506).

4. For a similar notion of the UN (as the “cathedral of the international community, the organizational repository of the community’s collective beliefs”) see Barnett (1997, 541).

5. Transforming leadership, then, implies a “metamorphosis” whereas “change” in the context of leadership simply means to substitute (Burns 2003, 24) and transforming the structure around it to infer roles.

6. Gordenker (1966/1967, 6) stressed this aspect at the example of Pope Paul VI and UNSG U Thant.

7. This is to ignore for now the fact that the pope is also head of a micro state.

8. Lord Gladwyn (1972, 257), first acting UNSG, pointed out this problem in attesting Hammarskjöld to be a leader but with the fault that he thought that he almost regarded himself as a Lay Pope. If he did, he should have realized that the Secretariat was not exactly the equivalent of a Papal Chancery. Even less was the General Assembly a College of Cardinals. He could not, in fact, have anything like the apparatus that is at the disposal of the Holy Father. Nor was there any generally acceptable philosophy which could illuminate his path. The Charter does not really indicate the things that states should do in order to achieve grace. It lays down rather what they should not do in order to avert disaster.

9. In the case of the pope this can also be located at the installment of like-minded cardinals to increase the chances that they will eventually elect a pope that continues their efforts (e.g. Goodstein, Pearce, and Pecanha 2016). This is different from the election of the UNSG which is predominantly a matter of the permanent members of the Security Council without any realistic chance of the office holder to influence the choosing of his successor.

10. One prominent output of the Pope’s engagement was the encyclical *Pacem in Terris* (John XXIII 1963, especially part III).

11. Here I refer to “institutions” as formal organizations with “prescribed hierarchies and the capacity for purposive action” (Keohane 1988, 384).

12. Griffith (2001) characterizes the ideal-type pope as one who “acts politically, but at the same time throws the politics of his acts into question.”

13. Again, the Pope does so, but insignificantly (i.e. for the State of the Vatican City).

14. Hammarskjöld captured this aspect in the following remarks: “The principles of the Charter are, by far, greater than the Organization in which they are embodied, and the aims which they are to safeguard are holier than the policies of any single nation or people” (Security Council Official Records 1956, 1–2).

15. This is also the case regarding religion within the UNO’s vast bureaucratic framework (e.g. Haynes 2014; Religion Counts 2002).

16. The Charter does not encompass a reference to God or religious values yet its Preamble makes it clear that “nations express their faith in the dignity and worth of the human person.”
